# Ketamine treatment for buprenorphine-precipitated opioid withdrawal: a case report

**DOI:** 10.3389/fpsyt.2025.1586945

**Published:** 2025-06-20

**Authors:** Ezioma Gbujie, Lisa Vercollone, Joji Suzuki

**Affiliations:** ^1^ Department of Psychiatry, Brigham and Women’s Hospital, Boston, MA, United States; ^2^ Department of Psychiatry, Harvard Medical School, Boston, MA, United States; ^3^ Department of Medicine, Brigham and Women’s Hospital, Boston, MA, United States

**Keywords:** buprenorphine, ketamine, buprenorphine opioid withdrawal, fentanyl, buprenorphine initiation

## Abstract

**Background:**

The presence of fentanyl in the drug supply is thought to contribute to the incidence of buprenorphine-precipitated opioid withdrawal (BPOW) during initiation. Long used as a surgical anesthetic and an analgesic, the utility of ketamine for psychiatric and substance use disorder indications continues to grow. We present a case of intravenous (IV) ketamine use on the general medical floor for the management of BPOW in a hospitalized patient.

**Case summary:**

A 72-year-old male presented to the emergency room with new-onset hematuria and was admitted for urological intervention. Hematuria was successfully managed with continuous bladder irrigation over 3 days. Unfortunately, on hospital day two, the patient developed myalgias, restlessness, and later revealed ongoing non-medical use of illicit opioids. The addiction consultation service was consulted on the second day of hospitalization and made a new diagnosis of severe opioid use disorder. Two separate attempts at buprenorphine/naloxone high-dose initiation to treat BPOW were not successful and the second was not responsive to standard symptomatic agents. An IV ketamine 27mg bolus was then administered, with an initial improvement followed by subsequent emergence delirium, which was addressed with IV haloperidol. The patient was eventually stabilized on buprenorphine/naloxone 8mg twice daily prior to discharge.

**Clinical significance:**

Ketamine may be an effective adjunctive agent in managing opioid withdrawal. Usually restricted to the intensive care unit and emergency department, this case report highlights both the potential and risks of IV ketamine on the general hospital floors. Further research is needed to better understand the safety of using ketamine to manage opioid withdrawal.

## Introduction

Fentanyl, a potent full mu-opioid receptor (henceforth MOR) agonist, has become prevalent in the non-medical opioid drug supply (1). Buprenorphine, a high-affinity partial MOR agonist, is one of three FDA-approved medications for the treatment of opioid use disorder (henceforth OUD) (2). Initiating buprenorphine as a treatment for illicit fentanyl use comes with the risk of precipitated opioid withdrawal (henceforth BPOW), thought to be due to the bioaccumulation of fentanyl and the high affinity but partial agonism of buprenorphine (3).

BPOW is defined as the onset of significant opioid withdrawal symptoms within 1 to 2 hours of buprenorphine dosing (4). A recent meta-analysis reported the incidence of BPOW in adults as ranging from 0% to 13.2% (5).

The risk for BPOW among those using fentanyl has led to the development of novel induction protocols, including low-dose, high-dose, and hybrid induction protocols (6). The low-dose protocol involves gradually up-titrating buprenorphine while the patient continues their usual full-agonist opioid, which can be tapered off or abruptly discontinued when the goal buprenorphine dose is attained (6). The high-dose protocol, in contrast, involves administering up to 32mg of buprenorphine to patients undergoing withdrawal within the first few hours (6). The hybrid protocol involves transitioning between the low and high dose as the clinical situation dictates (6).

Despite these newer protocols, the risk of BPOW remains. To add to the complexity, BPOW is a syndrome that is difficult to manage due to limited medication options. In addition to using the standard comfort medications to treat opioid withdrawal, both full MOR agonists and escalating doses of buprenorphine have been used with varying degrees of success (3). There is an active effort to explore the use of other medications already available clinically.

Recently, multiple case reports have been published detailing the use of ketamine as a rescue agent for severe BPOW, mostly in the emergency department (ED), intensive care, and outpatient settings (1, 7–10). Ketamine is an N-Methyl-D-Aspartate receptor (NMDA-receptor) antagonist that has been used as an anesthetic since it received FDA approval in 1970 (3). At anesthetic doses [intravenous (IV) 0.5–2mg/kg for induction of anesthesia or 0.25–0.35mg/kg followed by up to 1mg/kg/hr continuous infusion for maintenance of anesthesia (11)] it exhibits dissociative properties. However, at sub-anesthetic doses [0.25 to 0.5mg/kg bolus (max bolus: 35mg), followed by 0.05 to 0.25mg/kg/hour continuous infusion; titrated to pain goal and tolerability], ketamine exhibits potent analgesic properties that are not mediated via MOR activity, with a lower likelihood of eliciting dissociative properties (3). Its analgesic properties are thought to be centrally mediated through the inhibition of glutamate activation in the limbic system, hence reducing sensory processing (6).

The ameliorative effects of ketamine on BPOW are thought to be due to the synergistic potentiation of buprenorphine MOR signaling, reversal (re-sensitization) of fentanyl-induced MOR receptor desensitization, and inhibition of the descending pathways of hyperalgesia and central sensitization (3). Ketamine’s rapid antidepressant effects may also potentially address the depressive symptoms and subjective distress that often accompany BPOW (3). The doses of ketamine that have been used for BPOW are sub-anesthetic doses used for analgesia (3).

We present a case of BPOW in a hospitalized patient managed with IV ketamine on the general medical floor. The patient provided written consent to publish this case report.

## Case report

Mr. S is a 72-year-old male with a past medical history of benign prostatic hypertrophy, hypertension, a recent ankle fusion, and triple arthrodesis and a past psychiatric history of anxiety and attention deficit disorder who initially presented to the emergency room with 4 days of sudden-onset hematuria and lightheadedness. His only prescribed medications prior to presentation were tamsulosin 0.4mg and lisinopril 10mg daily. He was subsequently admitted to the general floors under urology care for manual and continuous bladder irrigation. Urine analysis was positive for an *Escherichia coli* urinary tract infection (UTI), which was treated with IV ceftriaxone.

On hospital day two, the patient reported myalgias, nausea, and restlessness, prompting a consultation request to the addiction consult service as the patient disclosed daily non-medical use of fentanyl (1.5 grams weekly) by insufflation for the prior 2 years, which he had initially started to manage pain related to a left foot fracture. He denied any history of overdoses, intravenous use, or prior use of medication for opioid use disorder (MOUD). He reported multiple unsuccessful self-driven attempts at cessation due to withdrawal symptoms and reported his last use of fentanyl as the day of admission. He met criteria for severe opioid use disorder based on The Diagnostic and Statistical Manual of Mental Disorders, Fifth Edition (DSM V) criteria because of the presence of craving, unsuccessful efforts to cut down use, use despite medical consequences, time spent obtaining and recovering, important activities given up due to use, tolerance, and withdrawal symptoms. An immunoassay urine toxicology obtained following his disclosure was positive only for fentanyl.

The patient expressed a desire to start treatment with buprenorphine/naloxone (henceforth buprenorphine for clarity). Because of the potential for imminent surgical urological intervention and the urology team’s preference, an initial decision to hold off buprenorphine initiation and manage his opioid withdrawal with full agonists and other comfort medications, including methocarbamol, clonidine, loperamide, dicyclomine, and ibuprofen, was made. Of note, due to the absence of further withdrawals, the patient did not require any of these in the following days before the induction.

**Figure 1:**
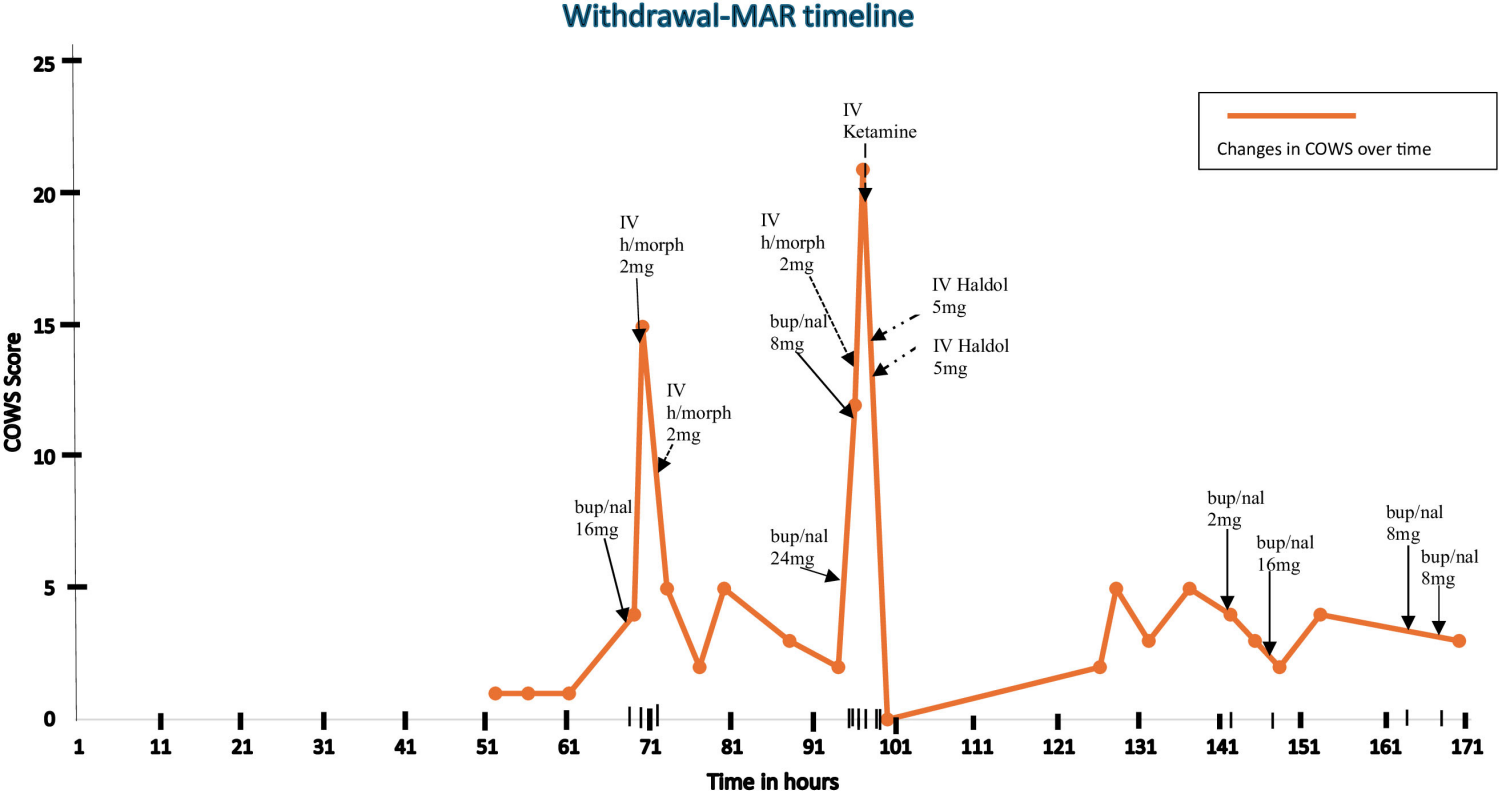
Withdrawal-MAR timeline.

On hospital day four, after Mr. S completed his IV antibiotics and the urology team ruled out the need for surgical intervention, the patient agreed to initiate buprenorphine using a macro-dose initiation protocol, stating a preference for the speediest initiation protocol to facilitate an early discharge. At this time, he was hemodynamically stable and remained cognitively intact. His clinical opiate withdrawal scale (henceforth COWS) score just prior to his first dose of buprenorphine was 1 (see **Figure 1**), and he had still not received any hospital-administered opioid or comfort medication prior to this time. Given the lack of opioid withdrawal and his reported last opioid use 4 days prior, it was thought that he would tolerate a high-dose initiation. The risks of opioid agonist treatment, including BPOW, were clearly discussed with the patient, and he agreed to start the initiation.

He received a single sublingual (SL) dose of 16mg buprenorphine (lower dose limit for high-dose initiation chosen due to age considerations) and within 30 minutes he developed nausea, agitation, tremulousness, diaphoresis, and diarrhea consistent with a COWS score of 15. A diagnosis of BPOW was made. The withdrawal symptoms were promptly managed with two successive doses of 2 mg IV hydromorphone, and his COWS score improved to 5 (see **Figure 1**). The patient at this time admitted to in-hospital use of his own fentanyl supply on the second day of his hospitalization. He was then transferred to the hospital’s medically managed inpatient unit, which provides a general floor level of care for further treatment. Further buprenorphine dosing was deferred to the following day.

On hospital day five, with a latest COWS score of 2, he was given 24 mg SL buprenorphine to complete the high-dose initiation that began the day prior. Within 2 hours, his COWS score increased to 12, consistent with BPOW. Attempts made to manage the BPOW with an additional dose of 8mg SL buprenorphine, 2mg IV hydromorphone, 2mg IV lorazepam, and 0.1mg oral clonidine were not successful as withdrawal symptoms worsened within 30 minutes to include worsened muscle cramping, anxiety, restlessness, and tachycardia. His COWS score increased to greater than 20 and his Richmond Agitation Scale Score (henceforth RASS) was noted to be +2. In lieu of a transfer to the ICU, a critical care nurse was invited to the general floor, who administered a one-time dose of IV ketamine 0.3mg/kg (27mg). This median dose from the sub-anesthetic range was chosen with consideration for the patient’s age. Within 10 minutes of administration, clinical symptoms resolved with RASS improving to −1.

Furthermore, 30 minutes after the administration of ketamine and the initial improvement, the patient developed acute delirium and hallucinations, which were managed with two doses of 5mg IV haloperidol 18 minutes apart. Given the presence of an altered mental status, an accurate COWS score could not be attained, however, a RASS of −1 was noted after haloperidol was administered. This event was thought to be emergence delirium due to the ketamine, especially in the setting of prior-administered psychoactive polypharmacy in an elderly patient.

By hospital day six, Mr. S’s mental status had returned to baseline. He acknowledged repeated use of non-prescribed fentanyl on hospital day four, which prompted a room search without yield.

Mr. S remained determined to be discharged on buprenorphine. On hospital day 7, the patient requested a test dose of 2 mg buprenorphine, which was well-tolerated. This was followed by 16 mg of buprenorphine 4 hours later, which was similarly well-tolerated. The decision to give 16 mg at this time relied on the hypothesis that Mr. S was likely stabilized on buprenorphine and the likelihood of further BPOW was thought to be low considering that Mr. S had tolerated a 2 mg dose on the same day, bringing the total administered buprenorphine to 50mg over 3 days, and buprenorphine’s long half-life.

On hospital day eight, Mr. S tolerated buprenorphine 8mg SL twice daily and was discharged thereafter on same regimen. He was referred to the hospital’s Bridge Clinic – a rapid access, low-barrier, low-threshold outpatient clinic for substance use disorders. At 180 days following discharge, Mr. S remains abstinent, connected to the Bridge Clinic, and maintained on buprenorphine 8mg twice daily.

## Discussion

The case reported here adds to the growing number of publications pointing to ketamine’s emerging role in managing opioid withdrawal during buprenorphine inductions. The rapid onset and efficacy in attenuating withdrawal symptoms make ketamine particularly suitable for use as an adjunctive treatment during BPOW. It has also been reported to facilitate tapering off opioids (12, 13). While symptomatic treatments such as clonidine are commonly recommended, ketamine may emerge as a more effective option in medical settings where ketamine is available and can be administered rapidly. Limitations, however, exist in the use of ketamine due to hospital policies that restrict the ordering only to those with privileges for approved indications in specific settings (7).

Several important lessons were learned from this case and are mentioned below.

The primary hypothesis for the etiology of recurrent BPOW in this patient is his use of non-prescribed fentanyl during the hospital stay. This highlights the need to remain aware of this possibility during hospitalizations, as the incidence of in-hospital use of illicit substances in patients with substance use disorders ranges from 34% to 40.5% (14, 15). The room search could have been done after the first BPOW incident.

The importance of accurately establishing the patient’s opioid status prior to buprenorphine induction cannot be overemphasized, and the withdrawal score tools, while not perfect, may be the most accurate guide. Mr. S was presumed to be in an opioid negative state at the time of the buprenorphine initiation despite the low COWS scores as he had been in the hospital for four days and had not received any hospital-administered opioids. Greenwald et al. posit in their working model that high-dose initiation protocols are best suited in opioid negative states, i.e., in opioid withdrawal, while low-dose initiations are best suited for opioid positive states, i.e., not in opioid withdrawal (6). When the clinical picture is conflicting, another consideration could be to have a clarifying discussion with the patient in a non-judgmental and non-punitive fashion, emphasizing the elevated risk of BPOW and other untoward interactions if buprenorphine is not introduced at the correct dose or time.

After the first BPOW, instead of attempting to surmount the withdrawal with more buprenorphine, our team could have considered switching to a low-dose initiation protocol using methadone as a bridge, since hybrid-inductions are also useful if the first approach fails. Another approach could have been the preemptive use of ketamine for the second attempt at high-dose initiation, as was described in Hailozian et al.’s case report (3).

It is unclear to what extent the patient’s age and polypharmacy burden prior to the administration of ketamine contributed to the adverse reaction the patient experienced. This provokes the consideration of age-related considerations/limitations in the doses of buprenorphine for high-dose initiations. Furthermore, in hindsight, optimizing the doses of fewer agents may have been a more advantageous approach.

A review of the literature appears to indicate that bolus doses of ketamine are initially administered for most indications, with an alternative to follow up with a continuous infusion (11). In this case, however, a continuous ketamine infusion on the general medical floors was not an alternative per hospital policy.

Ketamine re-emergence delirium commonly occurs at all ages and appears to be dose-dependent (11). A 2017 study by Avidan et al. demonstrated that a single sub-anesthetic dose of ketamine did not prevent post-operative delirium in the elderly and worsened the incidence of hallucinations and nightmares (16). Ketamine re-emergence delirium is thought to be mediated through agonism of dopamine D2 (henceforth DA-2) receptors (17). Risk factors include the use of higher doses, rapid IV administration, female sex, excessive stimulation during recovery, and a prior history of psychiatric disorders (11). Co-administration with benzodiazepines, which are Gamma-Aminobutyric acid A agonists (henceforth GABA_A_) or propofol (GABA_A_ agonist, DA-2 receptor antagonist), may reduce the risk (17). Agents such as haloperidol (DA-2 receptor antagonist), benzodiazepines (GABA_A_ agonist), and dexmedetomidine (alpha-2 adrenergic agonist) are used to manage ketamine re-emergence delirium so providers should ensure accessibility of these agents in clinical settings where ketamine is being administered (17, 18).

This case report hopes to add to the evidence base supporting the potential for ketamine in managing BPOW, while also highlighting the need to fine-tune approaches to mitigate inherent risks. Further research is needed to establish safer dosing and administration approaches that can expand the medical settings in which ketamine can be used for opioid withdrawal management.

## Data Availability

The raw data supporting the conclusions of this article will be made available by the authors, without undue reservation.
